# Comprehensive Molecular Diagnosis of Bardet-Biedl Syndrome by High-Throughput Targeted Exome Sequencing

**DOI:** 10.1371/journal.pone.0090599

**Published:** 2014-03-07

**Authors:** Dong-Jun Xing, Hong-Xing Zhang, Na Huang, Kun-Chao Wu, Xiu-Feng Huang, Fang Huang, Yi Tong, Chi-Pui Pang, Jia Qu, Zi-Bing Jin

**Affiliations:** 1 Division of Ophthalmic Genetics, Laboratory for Stem Cell and Retinal Regeneration, The Eye Hospital of Wenzhou Medical University, Wenzhou, China; 2 The State Key Laboratory Cultivation Base and Key Laboratory of Vision Science, Ministry of Health P. R. China, Wenzhou, China; 3 Jinan Eye Hospital, Second People’s Hospital, Jinan, China; 4 Fuzhou Southeastern Eye Hospital, Fuzhou, China; 5 Department of Ophthalmology and Visual Sciences, The Chinese University of Hong Kong, Hong Kong, China; Purdue University, United States of America

## Abstract

Bardet-Biedl syndrome (BBS) is an autosomal recessive disorder with significant genetic heterogeneity. BBS is linked to mutations in 17 genes, which contain more than 200 coding exons. Currently, BBS is diagnosed by direct DNA sequencing for mutations in these genes, which because of the large genomic screening region is both time-consuming and expensive. In order to develop a practical method for the clinic diagnosis of BBS, we have developed a high-throughput targeted exome sequencing (TES) for genetic diagnosis. Five typical BBS patients were recruited and screened for mutations in a total of 144 known genes responsible for inherited retinal diseases, a hallmark symptom of BBS. The genomic DNA of these patients and their families were subjected to high-throughput DNA re-sequencing. Deep bioinformatics analysis was carried out to filter the massive sequencing data, which were further confirmed through co-segregation analysis. TES successfully revealed mutations in BBS genes in each patient and family member. Six pathological mutations, including five novel mutations, were revealed in the genes *BBS2*, *MKKS*, *ARL6*, *MKS1*. This study represents the first report of targeted exome sequencing in BBS patients and demonstrates that high-throughput TES is an accurate and rapid method for the genetic diagnosis of BBS.

## Introduction

**Figure 1:**
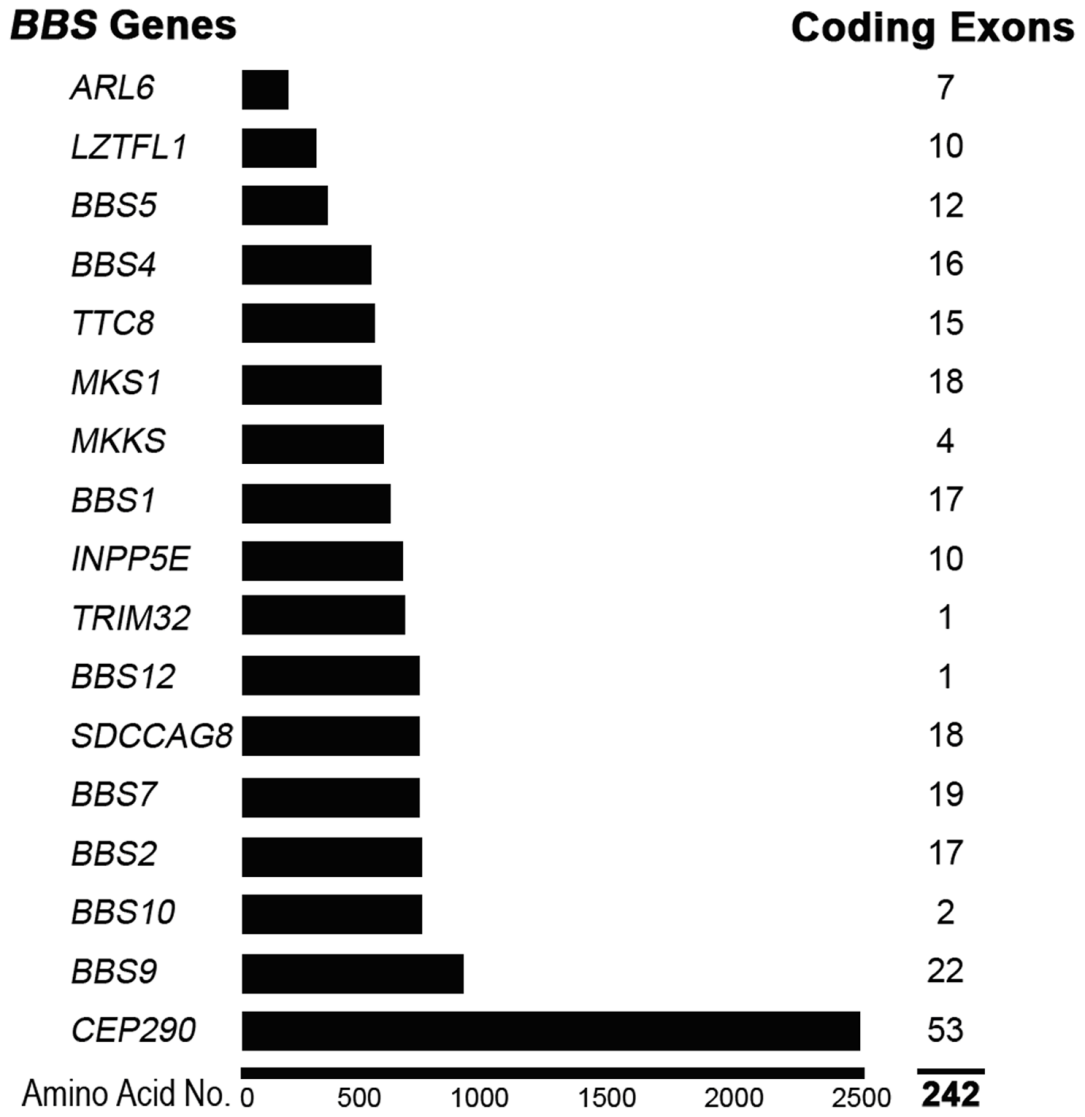
Causative Genes of Bardet-Biedl Syndrome. Seventeen *BBS* genes have been mapped and identified, including a total of 242 coding exons.

Bardet-Biedl syndrome (BBS) is a pleiotropic autosomal recessive disorder. The main characteristics of BBS are retinitis pigmentosa, obesity, polydactyly, hypogenitalism, renal anomalies, and learning difficulties [1], [2]. However other clinical features including mild developmental delay, speech delay, congenital heart disease, poor coordination, and an increased incidence of diabetes mellitus and hypertension have been reported in BBS patients [3]–[5]. Presently, heterozygous carriers of BBS are screened for mutations in BBS genes by direct DNA sequencing. A total of 17 genes linked to BBS have identified so far (Figure 1), which contain a total of 242 coding fragments. This large number of genes makes diagnosis of BBS by current methods of DNA sequencing both time-consuming and costly. Thus there is an urgent need to develop a more efficient method to screen carriers of this disease.

Targeted exome sequencing (TES) has been shown to be more efficient than traditional sequencing in the discovery of novel disorder-related genes or mutations in large genomic regions [6]–[9]. Therefore, TES be advantageous in comprehensively screening heterozygous carriers for a panel of known BBS genes. In this study, we developed an efficient strategy for full-scale molecular screening of BBS genes using high-throughput TES.

## Materials and Methods

### Patient Recruitment

This study was approved by the Ethics Committee of the Eye Hospital of Wenzhou Medical University (No.KYK2012-7), and adhered to the rules of the Declaration of Helsinki. Written informed consent was obtained from all study participants. The study participants consisted of five unrelated patients, their unaffected relatives, and a panel of 300 healthy controls. BBS was diagnosed primarily by retinitis pigmentosa, polydactyly, obesity, hypogenitalism, cognitive impairment, and delayed development of motor skills. Detailed medical and family histories were obtained by personal interviews with patients and their family members. Comprehensive ophthalmic examinations, including perimetry and fundus photography, were carried out in all patients. Peripheral venous blood samples were obtained from all study participants.

### Targeted Capture Preparation, Sequencing and Bioinformatics Analysis

Genomic DNA was extracted from blood lymphocytes using a DNA Extraction kit (TIANGEN, Beijing) according to the manufacturer’s instructions. Briefly, DNA was sheared mechanically with Nanodrop 2000 (Thermal Fisher Scientific, DE). DNA (<3 μg) was prepared for the indexed Illumina libraries based on manufacturer’s request. Library size including adapter was sheared to 350–450 bp. The coding exons and flanking regions of 144 genes related to inherited retinal diseases (Table S1) were selected and captured using a GenCap custom enrichment kit (MyGenostics, Beijing) as previously described [10], [11]. Genomic DNA from proband was fragmented and then mixed with GenCap probe (MyGenostics, Beijing) for PCR and hybridization. Sample was washed by MyOne beads (Life Technology) and resuspended in binding buffer. The mixed sample was transferred with MyOne beads, and rotated for 1 hour on a rotator. Finally, DNA was eluted with Buffer Elute and amplified in post-capture. The enriched libraries were subjected to sequencing on IlluminaSolexaHiSeq 2000 sequencer. High-quality sequencing depth was mapped for all targeted regions, which count for approximately 98.5% on 144 genes. The low quality reads and adaptor sequences were filtered out with the cutadapt program and the Solexa QA package [12]. Picard program [13] was used to remove the PCR duplicates. After high-quality reads were retrieved, the clean reads were aligned using SOAPaligner program [14] according to human genome parameters (hg19). Subsequently, we determined SNPs using the SOAPsnp program, realigned the reads with BWA, and detected the deletions or insertion (InDels) with the GATK software [15]. After annotation of the identified SNPs and InDels with the Exome-assistant program (http://122.228.158.106/exomeassistant), the short read alignment, candidate SNPs, and InDels were viewed by Magic Viewer [16]. Finally, variant-special information was collected and predicted for pathogenicity using four algorithms, PolyPhen, SIFT, Mutationtaster and PMut [17]. Sequencing data were deposited in NIH Short Read Archive (SRP033329).

### Expanded Validation

Genomic DNA from the probands of the two families were subjected to the same capture targeted exome sequencing and analysis. Filtered candidate variants were confirmed by Sanger sequencing. The coding exons that contain the detected mutations were amplified with Ex Taq DNA polymerase (Takara, Dalian). PCR samples were visualized on agarose gels, purified, and sequenced on an ABI 3500 Genetic Analyzer (Applied Biosystems, CA). Sequence traces were analyzed using the Mutation Surveyor (Softgenetics, PA). The mutations in the proband were confirmed DNA from their family members by the same procedure. The *BBS2* c.563C>T mutation results in loss of the recognition site of restrictionenzymeEcoRV (NEB, Beijing), which was finally re-confirmed using Restriction Fragment Length Polymorphism Method (RFLP). The coding exon and flanking region were amplified by PCR. After digestion with restrictionenzymeEcoRV, the mutated PCR sample showed different bands in agarose gel electrophoresis compared with that of the wild type. All genomic DNA samples were collected upon informed consent.

## Results

### Phenotypic Characterization of Probands

**Figure 2:**
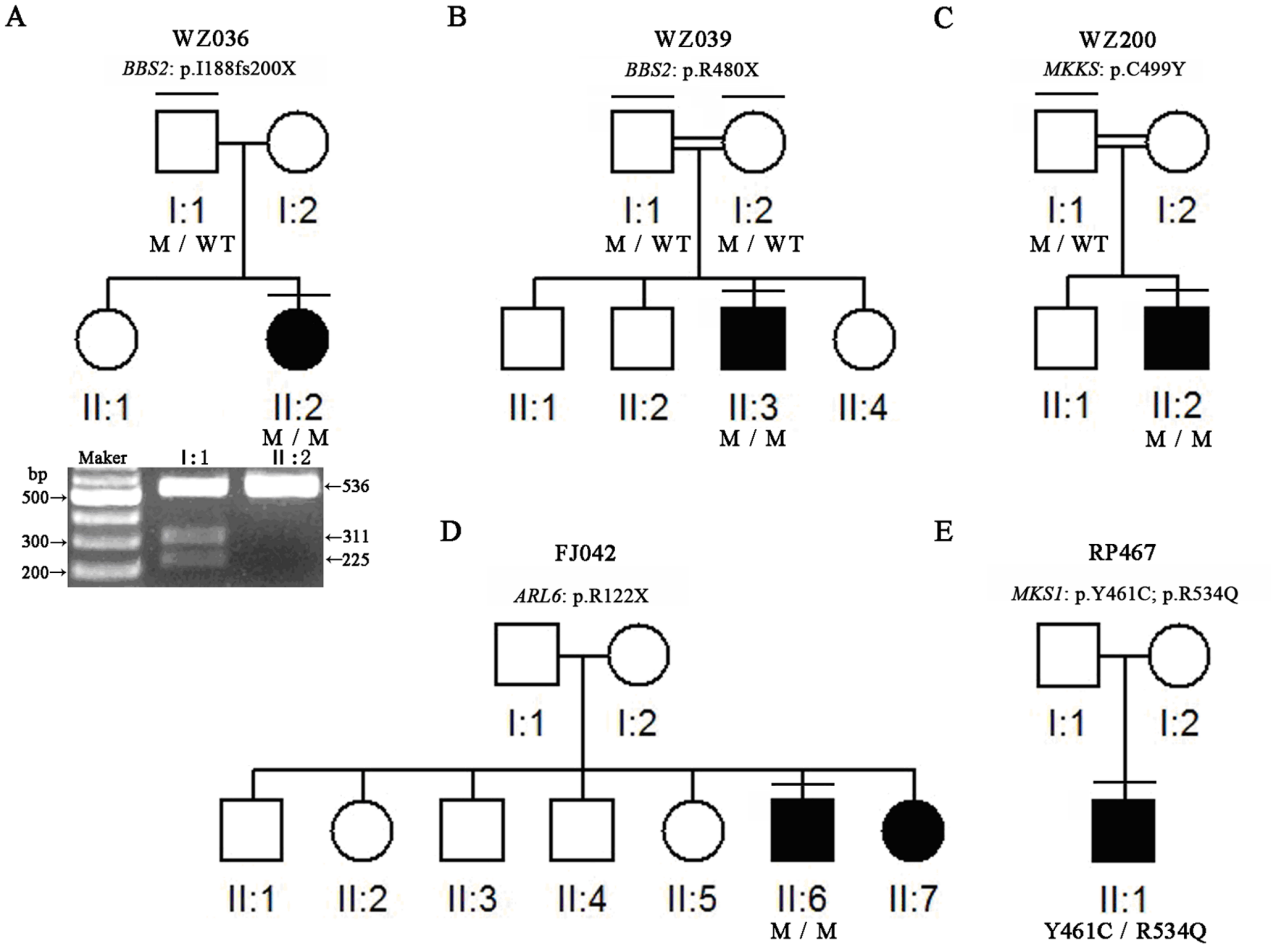
Five BBS Pedigrees underwent Comprehensive Mutational Screening. The pedigrees of five families with Bardet-beidl syndrome (BBS) are shown. A: In pedigree of WZ036, mutation c.563C>T was re-confirmed by restriction fragment length polymorphism method with restrictionenzymeEcoRV. The homozygous sample (WZ036-I:2) was not digested, while the heterozygous sample (WZ036-I:1) was partially digested. B, C, D: *BBS* genes mutations were detected by TES, and confirmed by direct sequencing with intra-familiar members. Squares indicate males; circles indicate females; solid symbols indicate affected; open symbols indicate unaffected; Bar on the symbol indicates the proband examined by TES; WT, wildtype; M indicates mutation.

Each BBS patient in this study displayed typical symptoms such as retinitis pigmentosa, obesity, and polydactyly [5], [18]. Additionally, affected patients displayed typical fundus of bone-spicule hyperpigmentation and attenuated arteries. One child proband (WZ036-II:2) complained of progressive night blindness and impaired visual acuity (Figure 2A). Similarly, two other probands (WZ039-II:3 and WZ200-II:2) experienced night blindness as early as childhood, in addition to having been born with an extra-digit on their foot.. Following detailed personal interviews with patients and their unaffected family members, we found that the probands showed other signs of speech delay and poor coordination. Notably, one proband (WZ039-II:3) has not been able to father children, indicating a sign of genital anomalies (Figure 2B). Other BBS symptoms were observed in patients such as high blood pressure and delayed ability in learning to walk. Taken together, these clinical symptoms and signs suggested the diagnosis of BBS.

### TES and Cosegregation Analysis Revealed Causative Mutations

The genomic DNA of all five probands was subjected to targeted exome sequencing (TES). 144 genes related to inherited retinal degeneration were analyzed including BBS and retinitis pigmentosa (RP). Coverage for targeted regions for each sample was more than 96.5% with an average sequencing depth of 133.8 on the targeted genomic regions. Targeted exons covering greater than 10 reads and greater than 20 reads ranged from 80.6% to 99.5% and 63.7% to 99.2%, respectively. Sequence quality of all known BBS genes was measured (Table S2). Finally, more than 140 variants were detected for each sample. The variants were filtered by those that had been reported in the HapMap 28. Then, SNPs with a MAF>0.05 in 1000 Genome Project were removed from analysis [19], leaving approximately 10–21 variations that satisfied these criteria. Further analyses with PolyPhen, SIFT, PANTHER and Pmut demonstrated that presence of these variants predict pathogenicity of BBS in patients [17]. Following this step-by-step filtering protocol, six mutations in *BBS2*, *MKKS*, *ARL6*, *MKS1* genes were finalized (Table 1). Population frequency and computational assessment of each missense mutation were also determined (Table S3 and Table S4).

**Table 1:** Identified Mutations in *BBS* Genes.

Family	Subject	Phenotype	Gene	Mutation	Type	Amino acid	Reported
WZ036	II:2(Proband)	+	*BBS2*	c.563delT	homo	p.I188fs,200X	Novel
	I:1(Father)	–	*BBS2*	c.563delT	hetero	p.I188fs,200X	Novel
WZ039	II:3(Proband)	+	*BBS2*	c.1438C>T	homo	p.R480X	Novel
	I:1(Father)	–	*BBS2*	c.1438C>T	hetero	p.R480X	Novel
	I:2(Mother)	–	*BBS2*	c.1438C>T	hetero	p.R480X	Novel
WZ200	II:2(Proband)	+	*MKKS*	c.1496G>A	homo	p.C499Y	Novel
	I:1(Father)	–	*MKKS*	c.1496G>A	hetero	p.C499Y	Novel
FJ042	II:6(Proband)	+	*ARL6*	c.364C>T	homo	p.R122X	Reported
RP467	II:1(Proband)	+	*MKS1*	c.1382A>G	hetero	p.Y461C	Novel
			*MKS1*	c.1601G>A	hetero	p.R534Q	Novel

**Figure 3:**
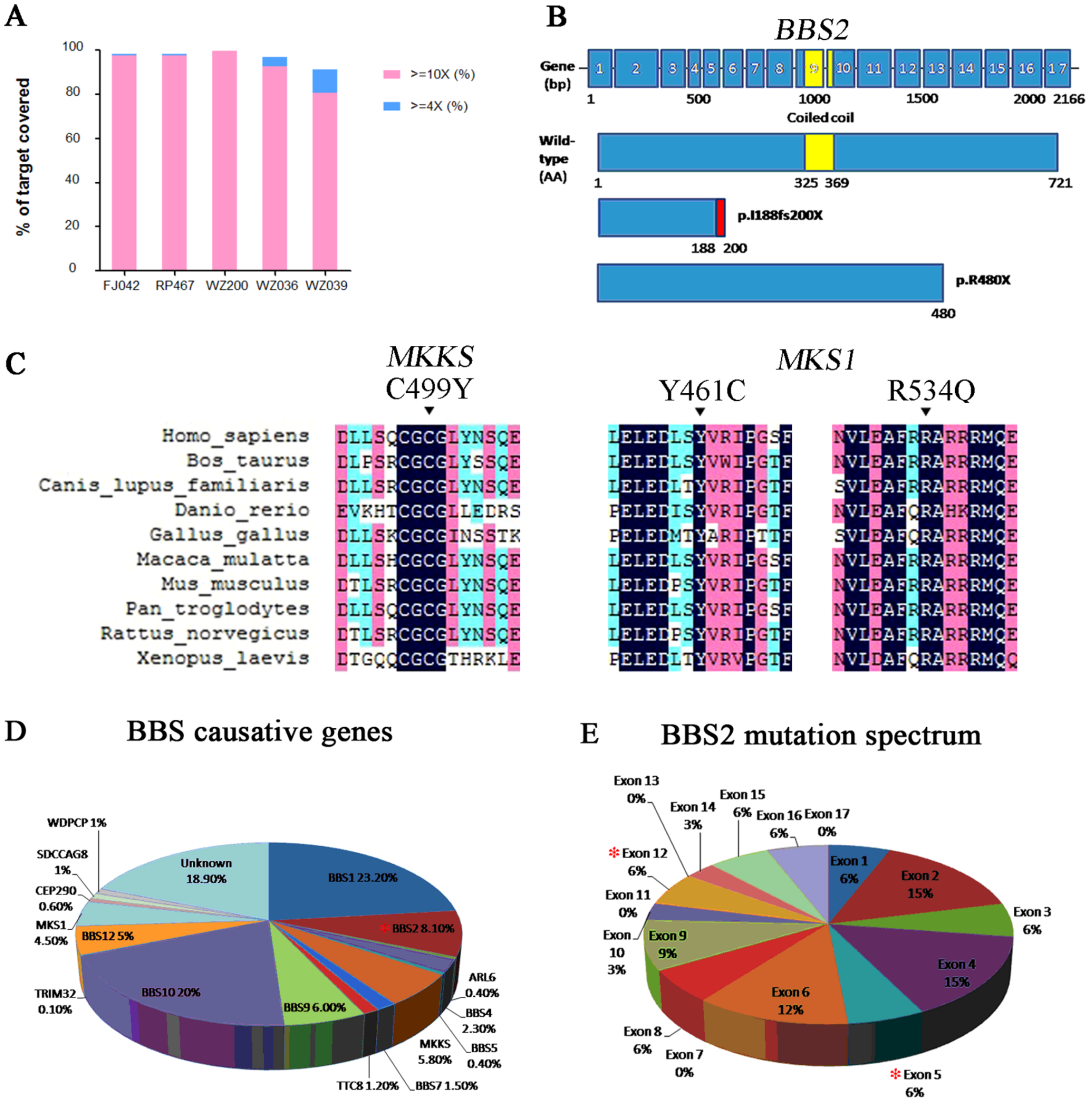
Conservation of Missense Mutations in BBS Genes. A: Coverage of the reads > 10 and > 4 in each sample; B: Gene and protein structures of *BBS2*, including p.I188fs200X and p.R480X..The yellow region indicates the coiled coil domain; the blue indicates the flanking peptide chain region; the red box represents the frame shift peptide chain (p.I188fs200X). C: All the missense mutations of BBS genes were located within a highly conserved region compared to different species. D: Seventeen genes were mapped and identified with BBS so far, in which patients with *BBS1* or *BBS10* accounts for more than 20% respectively. E: More than 10% reported mutations of *BBS2* are located at exon 2, 4, 6, while the two novel mutations discovered in this study were located at exon 5 and 12. Asterisk represents the locations of mutations.

To confirm the TES results, we performed Sanger sequencing to validate the mutations in each BBS family. In family WZ036, the homozygous mutation of c.563delT (p.I188fs200X) was confirmed. Meanwhile, the unaffected father in this family had a heterozygous *BBS2* mutation of c.563delT (Table 1). The mutation was re-confirmed by restriction fragment length polymorphism (RFLP) (Figure 2A). Since the parents (WZ036-I:1 and WZ036-I:2) were phenotypically normal, it is thus conceivable that each of the parents carried a heterozygous mutation in BBS2 and transmitted to WZ036-II:2 (Figure 2A). The family WZ039 showed the same genetic continuity (Figure 2B). The proband (WZ039-II:3) was found to carry a homozygous mutation (c.1438C>T) in *BBS2* as well as two heterozygous variations (c.1966G>A, p.D656N; c.2350C>T, p.R784C) in *BBS9*. Each of the proband’s unaffected parents had a c.1438C>T heterozygous mutation in *BBS2*. Therefore, both parents must have transmitted the c.1438C>T mutation of *BBS2* to WZ039-II:3 (Table 1). Meanwhile, the unaffected father in family WZ039 harbored both c.1966G>A and c.2350C>T heterozygous variations of *BBS9*.The BBS2 mutations, c.563delT and c.1438C>T, were both predicted to create a premature stop codon. Furthermore, the c.563delT (p.I188fs200X) mutation leads to loss of the coiled coil regions (Figure 3B) [20]. Through intra-familial analysis, two homozygous mutations, c.1496G>A (p.C499Y) in *MKKS* and c.364C>T (p.R122X) in *ARL6*, were confirmed in pedigrees WZ200 and FJ042. Additionally, compound heterozygous *MKS1* mutations (c.1382A>G, c.1601G>A) were revealed in pedigree RP467 (Table 1). This is the first reporting of five out of the 6 BBS mutations identified, which may indicate an ethinic difference of the mutation spectrum in the Chinese population. In addition to the nonsense or frameshift mutations identified in this study, three missense mutations were predicted to alter highly conserved amino acid residues (Figure 3C). In addition, all identified mutations were absent in the control participants. These results collectively demonstrate that genetic defects in BBS can be successfully identified through comprehensive molecular screening using TES.

## Discussion

Bardet-Biedl syndrome (BBS) is a rare inherited autosomal recessive disorder [4], which ranges in prevelence from 1∶13,500 to 1∶160,000 in various populations [4], [21], [22]. In this study, we identified 17 loss-of-function mutations in BBS genes that can accurately diagnose the disease. Presently, genetic diagnosis of BBS depends on the identification of mutations in *BBS* genes by conventional DNA sequencing, which because of the great breadth of genes and coding regions, is time-consuming and expensive. In this study, we demonstrate that targeted exome sequencing (TES) is a highly efficient and practical method for genetic diagnosis of BBS through the screening of 144 causative genes of inherited retinal degeneration in BBS patients and their unaffected (carrier) family members. Our results show that TES can provide more complete genetic information because it screens alterations in more than one hundred genes simultaneously. This ability to screen multiple genes at once is important as studies have shown that BBS is a pleiotropic disease and therefore some patients may harbor mutations in multiple genes [1], [2], [23]. Additionally, our TES protocol requires only one genetic sample from a proband, which is less that required by traditional Sanger sequencing to detect mutations of a candidate gene. The method developed in the present study would be sufficient for comprehensive molecular screening of BBS syndrome.

Previous studies indicate that mutations in *BBS1* and *BBS10* mutations dominantly predispose patients to the disorder (Figure 3D) [4]. However, Chinese BBS patients were reported to harbor mutations in *BBS7* [24], [25]. Meanwhile, our data show that presence of a *BBS2* mutation led to disease in two Chinese families, suggesting there might be a different *BBS* gene mutation spectrum among distinct populations (Figure 3E). Among the six mutations identified in this study, five mutations have not been previously reported. This result supports the hypothesis that there is a distinct BBS mutation spectrum in different populations. In this study, one family (WZ039) was found to carry a nonsense mutation and two missense variations in *BBS2* and *BBS9* genes respectively. As previous reports of digenic mutations in BBS patients [26] and both BBS2 and BBS9 are parts of a stable multi-protein complex known as the BBSome [27], the pathogenicity of these two variations in *BBS9* are questionable and further functional experiments are needed to elucidate the findings.

In summary, we have shown that TES can be used to efficiently and accurately diagnosis BBS in both patients and their unaffected family members. In addition, we have discovered five novel mutations, which expand the current known BBS mutation spectrum.

## Supporting Information

Table S1 **List of the 144 genes captured in this study.**
(DOC)

Table S2 **Sequence coverage for all known BBS genes.**
(DOC)

Table S3 **Population frequency of the variants.**
(DOC)

Table S4 **Computational assessment of the missense mutations.**
(DOC)
