# Supplementary material for: Comprehensive Molecular Diagnosis of Bardet-Biedl Syndrome by High-Throughput Targeted Exome Sequencing
Source: PLoS One. 2014 Mar 7;9(3):e90599. doi: 10.1371/journal.pone.0090599 (PMC3946549; doi:10.1371/journal.pone.0090599)
Supplement: Table S2 — Sequence coverage for all known BBS genes. (DOC) [file pone.0090599.s002.doc]

**Table S2. Sequence coverage for all known BBS genes.**

| ***Sample*** | ***Aligned (%)*** | ***Coverage*** | ***Average***  ***depth*** | ***Coverage >4X*** | ***Coverage >10X*** | ***Coverage >20X*** |
| --- | --- | --- | --- | --- | --- | --- |
| FJ042 | 99.53 | 98.30% | 149.45 | 97.70% | 97.50% | 96.60% |
| WZ036 | 98.28 | 71.80% | 35.54 | 62.70% | 54.90% | 45.90% |
| RP467 | 99.52 | 98.20% | 161.03 | 97.80% | 97.50% | 96.70% |
| WZ200 | 99.62 | 98.30% | 300.13 | 97.70% | 97.20% | 96.90% |
| WZ039 | 99.38 | 78.30% | 51.8 | 66.30% | 59.00% | 51.60% |
