# Supplementary material for: Comprehensive Molecular Diagnosis of Bardet-Biedl Syndrome by High-Throughput Targeted Exome Sequencing
Source: PLoS One. 2014 Mar 7;9(3):e90599. doi: 10.1371/journal.pone.0090599 (PMC3946549; doi:10.1371/journal.pone.0090599)
Supplement: Table S3 — Population frequency of the variants. (DOC) [file pone.0090599.s003.doc]

**Table S3. Population frequency** of the variants.

| ***Gene*** | ***Mutation*** | ***Protein*** | ***Database*** | | |
| --- | --- | --- | --- | --- | --- |
|  |  |  | **Snp137** | **1000 Genome** | **ESP6500** |
| *MKKS* | c.1496G>A | p.C499Y | Novel | Novel | Novel |
| *MKS1* | c.1382A>G | p.Y461C | Novel | Novel | Novel |
| *MKS1* | c.1601G>A | p.R534Q | rs199910690 | 0.0005 | Novel |
| *ARL6* | c.364C>T | R122X | Novel | Novel | Novel |
| *BBS2* | c.1438C>T | p.R480X | Novel | Novel | Novel |
| *BBS2* | c.563delT | p.I188fs200X | Novel | Novel | Novel |
