# Supplementary material for: Comprehensive Molecular Diagnosis of Bardet-Biedl Syndrome by High-Throughput Targeted Exome Sequencing
Source: PLoS One. 2014 Mar 7;9(3):e90599. doi: 10.1371/journal.pone.0090599 (PMC3946549; doi:10.1371/journal.pone.0090599)
Supplement: Table S4 — Computational assessment of the missense mutations. (DOC) [file pone.0090599.s004.doc]

**Table S4. Computational assessment of the missense mutations.**

| ***Gene*** | ***Mutation*** | ***Protein*** | ***Mutationtaster*** | ***Polyphen2*** | ***SIFT*** | ***PMut*** |
| --- | --- | --- | --- | --- | --- | --- |
| *MKKS* | c.1496G>A | p.C499Y | Disease causing | Probably damaging | Tolerated | Pathological |
| *MKS1* | c.1382A>G | p.Y461C | Disease causing | Probably damaging | Damaging | Pathological |
| *MKS1* | c.1601G>A | p.R534Q | Disease causing | Probably damaging | Tolerated | Pathological |
